# Admittance Investigation of MIS Structures with HgTe-Based Single Quantum Wells

**DOI:** 10.1186/s11671-016-1276-1

**Published:** 2016-02-01

**Authors:** Ihor I. Izhnin, Sergey N. Nesmelov, Stanislav M. Dzyadukh, Alexander V. Voitsekhovskii, Dmitry I. Gorn, Sergey A. Dvoretsky, Nikolaj N. Mikhailov

**Affiliations:** Scientific Research Company “Caratt”, Striskaya St. 202, Lviv, 79031 Ukraine; National Research Tomsk State University, Lenina Av. 36, 634050 Tomsk, Russia; A.V. Rzhanov Institute of Semiconductor Physics SB RAS, akademika Lavrent`eva Av. 13, 630090 Novosibirsk, Russia

**Keywords:** HgCdTe, Molecular beam epitaxy, Single quantum well, Admittance, 73.40.Qv, 72.20.-i, 73.63.Hs

## Abstract

This work presents results of the investigation of admittance of metal-insulator-semiconductor structure based on Hg_1 − *x*_Cd_*x*_Te grown by molecular beam epitaxy. The structure contains a single quantum well Hg_0.35_Cd_0.65_Te/HgTe/Hg_0.35_Cd_0.65_Te with thickness of 5.6 nm in the sub-surface layer of the semiconductor. Both the conductance-voltage and capacitance-voltage characteristics show strong oscillations when the metal-insulator-semiconductor (MIS) structure with a single quantum well based on HgTe is biased into the strong inversion mode. Also, oscillations on the voltage dependencies of differential resistance of the space charge region were observed. These oscillations were related to the recharging of quantum levels in HgTe.

## Background

In the last few years, admittance spectroscopy has been extensively used to obtain the information about some properties of semiconductor heterostructures with quantum wells [1, 2] and quantum dots [3–5], particularly with use of metal-insulator-semiconductor (MIS) structures based on this material. It should be noted that studies of the characteristics of MIS structures based on HgCdTe with inhomogeneous composition distribution are still extremely rare. The influence of near-surface graded-gap layers on the admittance of MIS structures based on molecular beam epitaxy-grown HgCdTe was studied in detail in [6, 7]. The properties of a MIS structure based on molecular beam epitaxy (MBE)-grown *n*-Hg_0.75_Cd_0.25_Te material comprising CdTe/HgTe superlattice in the sub-surface region of a heterostructure were experimentally investigated in [8]. It was shown that at high hole concentration (*p* > 10^17^ cm^−3^) in *p*-HgCdTe, quantization of state density in the inversion layer was possible, which led to quantum oscillations in the strong inversion mode of photo-electromotive force [9], capacitance, and conductance [10].

This paper presents the results of experimental studies of the effect of a single quantum well based on HgTe induced into *p*-HgCdTe material on the admittance of the MIS structures in wide frequency and temperature ranges.

## Presentation of the Hypothesis

The hypothesis is that the MIS structures based on *p*-HgCdTe biased in the strong inversion mode can be used for the characterization of a narrow single quantum well (SQW) based on HgTe in the near-surface layer of the semiconductor using measurements of admittance.

## Testing the Hypothesis

**Fig. 1:**
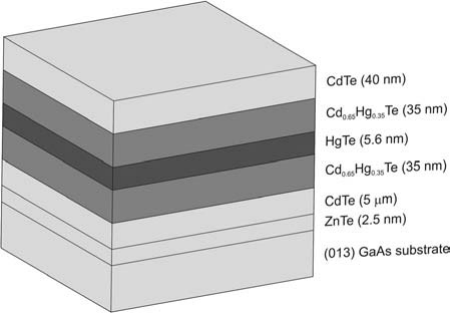
Scheme of the layer position in the structure with a SQW

The investigated samples were grown by MBE method on GaAs (013) substrates with ZnTe (~2.5-nm thickness)/CdTe (~5-μm thickness) buffer layers at the A.V. Rzhanov Institute of Semiconductor Physics (Novosibirsk, Russia). In a typical nanostructure, a HgTe single quantum well with the width *d*_*w*_ = 5.6 nm was sandwiched between two Hg_1 − *x*_Cd_*x*_Te (*x* = 0.65) barrier layers with the thickness *d*_*b*_ = 35 nm (see Fig. 1). Then, the 40-nm-thick CdTe layer was grown upon the upper barrier layer. A double-layer insulator SiO_2_/Si_3_N_4_ was deposited over the top CdTe layer. For comparison, some of the investigated structures did not include a SQW. Measurements were carried out with the use of an automated admittance spectroscopy installation based on non-optical Janis cryostat and Agilent E4980A immittance meter.

**Fig. 2:**
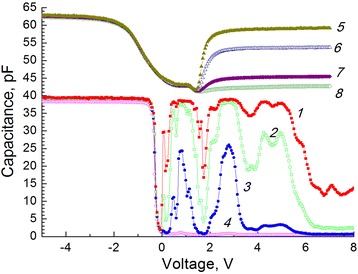
CV characteristics of structures with a SQW (*1*–*4*) and without a SQW (*5*–*8*) measured at various frequencies, kHz: *1*—1, *2*—3, *3*—15, *4*—150, *5*—10, *6*—20, *7*—50, and *8*—100

Figure 2 shows dependencies of capacitance on voltage for the MIS structure with a HgTe SQW measured at a temperature 9 K for various frequencies of the test signal. This figure also shows capacitance-voltage (CV) characteristics for MIS structures based on *p*-Hg_0.78_Cd_0.22_Te without a SQW measured at a temperature 20 K. In Fig. 2, it can be seen that HgCdTe has *p*-type conductivity and in the strong inversion mode at frequencies from 1 to 150 kHz, non-monotonic change in capacitance is observed. Capacitance and conductance maxima are observed at the same bias voltages. At low frequency, capacitance-voltage characteristic has low-frequency behavior and maxima become less pronounced than at frequencies 1–40 kHz. At 150 kHz, CV characteristic demonstrates high-frequency behavior and capacitance maxima also appear less clearly.

**Fig. 3:**
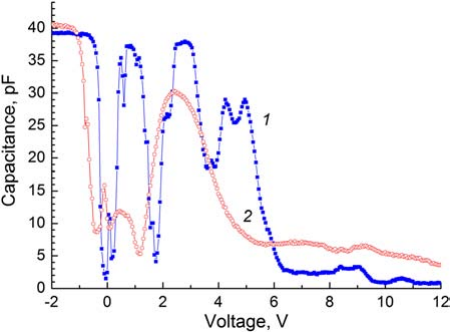
CV characteristics of a structure with a HgTe SQW measured at a frequency of 3 kHz for various temperatures, K: *1*—9 and *2*—70

**Fig. 4:**
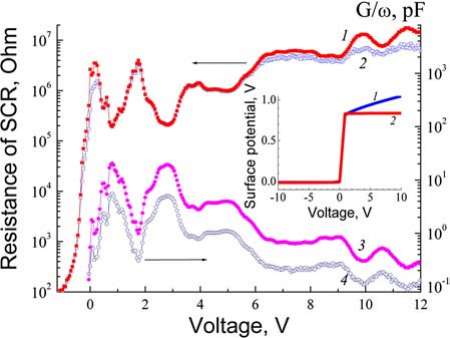
SCR differential resistance dependencies and normalized conductance versus voltage bias measured at various frequencies, kHz: *1* and *3*—40 and 2 and 4—150. The *inset* shows the dependencies of the surface potential on voltage calculated taking into account the effects of degeneracy and non-parabolicity (*1*) and not taking into account these effects (*2*)

Figure 3 shows CV characteristic of the MIS structure with a SQW measured at 3 kHz at temperatures 9 and 70 K. It is seen that at 70 K, maxima become diffused and are pronounced quite weakly. Figure 4 shows voltage dependencies of differential resistance of the space charge region (SCR) for the structure with a SQW measured at 40 and 150 kHz at 9 K. Also, this figure shows a normalized CV characteristic measured at the same conditions. It can be seen that conductance maxima and minima of the differential resistance of the SCR are observed at the same values of the bias. For structures without a HgTe SQW, non-monotonic changes of capacitance and conductance in the strong inversion mode were not observed.

We will briefly describe the most probable mechanism for the appearance of the maxima at CV characteristics in the strong inversion mode for the MIS structure comprising a HgTe SQW. The decrease of the differential resistance of the SCR is related to the increase in the flow of minority charge carriers due to their emission from a SQW. The potential in the region of a SQW for *p*-HgCdTe depends on the bias voltage in the strong inversion because of the effects of degeneracy and conduction band non-parabolicity. When the Fermi level in the SQW region approaches the level of quantization, quantization level recharges following a change of the test signal (possibly due to trap-assisted tunneling transitions). Capacitance of dimensional quantization level in a SQW contributes to the full capacitance of the structure and appears at intermediate frequencies in the strong inversion. When CV characteristic has low-frequency behavior, capacitance of the MIS structure in the strong inversion tends to the insulator capacitance and maxima associated with dimensional quantization levels do not appear. When CV characteristic has high-frequency behavior, the carrier concentration in the inversion layer does not have enough time to follow the test signal and contribution of the capacitance of quantization levels to the full capacitance decreases. Since the charge carrier thermal energy increases with the increase in temperature, dimensional quantization effects are weak.

Energies of dimensional quantization levels in a quantum well can be approximately defined. To do this, it is necessary to define the flat-band voltage in the MIS structure comprising a SQW in terms of flat-band capacitance value and to obtain surface potentials corresponding to maxima of capacitance in the strong inversion. Then, it is necessary to build the potential spatial distribution in the sub-surface layer and to find the potential under the given bias voltage in a SQW. Knowing the position of the Fermi level in the flat-band mode, the position of dimensional quantization levels can be found relative to the allowed energy bands in HgCdTe barriers.

## Implications of the Hypothesis

It is experimentally shown that for MIS structures based on MBE *p*-HgCdTe, the presence of a SQW with thickness of 5.6 nm can lead to the appearance of capacitance-voltage and conductance-voltage characteristic peaks in the strong inversion mode (and minima on voltage dependencies of differential resistance of the SCR). Capacitance maxima in the strong inversion mode are observed in an intermediate case, i.e., between the low-frequency and high-frequency behavior. It is shown that when considering the effects of degeneracy and non-parabolicity of the conduction band, the surface potential at strong inversion depends on the bias voltage [11]. The calculated dependencies of the surface potential on voltage at various approximations are shown in the inset in Fig. 4. It is assumed that the dimensional quantization level recharge capacitance in a SQW contributes to the overall capacitance of the structure in the strong inversion. An approximate method for determining the energy levels of quantum wells with the use of data obtained from the CV characteristic measurements is proposed. Then, the first two maxima of capacitance for the MIS structure with a SQW in the strong inversion mode correspond to energies *E*_c_ = 0.520 eV and *E*_c_ = 0.210 eV, respectively. Here, *E*_c_ is the energy of conduction band edge of the barrier layer near the border with well. The calculation showed that the first two maxima of capacitance observed on the CV characteristics in the experiment may be associated with the presence of dimensional quantization levels.

## Abbreviations

CV: capacitance-voltage
MBE: molecular beam epitaxy
MIS: metal-insulator-semiconductor
SCR: space charge region
SQW: single quantum well

## References

[1] Brounkov PN, Benyattou T, Guillot G, Clark CA (1995). Admittance spectroscopy of InAlAs/InGaAs single-quantum-well structure with high concentration of electron traps in InAlAs layers. J Appl Phys.

[2] Zubkov VI (2007) Characterization of In_*x*_Ga_1-*x*_As/GaAs quantum-well heterostructures by CV measurements: band offsets, quantum-confinement levels, and wave functions. Semiconductors 41:320–326

[3] Zhang SK, Zhu HJ, Lu F, Jiang ZM, Xun W (1998). Coulomb charging effects in self-assembled Ge quantum dots studied by admittance spectroscopy. Phys Rev Lett.

[4] Yakimov AI, Dvurechenskii AV, Nikiforov AI, Pchelyakov OP (1998). Formation of zero-dimensional hole states in Ge/Si heterostructures probed with capacitance spectroscopy. Thin Solid Films.

[5] Yakimov AI, Dvurechenskii AV, Nikiforov AI, Bloshkin AA, Nenashev AV, Volodin VA (2006). Electronic states in Ge∕Si quantum dots with type-II band alignment initiated by space-charge spectroscopy. Phys Rev B.

[6] Voitsekhovskii A, Nesmelov S, Dzyadukh S (2014) Influence of composition of the near-surface graded-gap layer on the admittance of metal-insulator-semiconductor structures based on graded-gap MBE *n*-Hg_1−x_Cd_x_Te in wide temperature range. Opto-Electron Rev 22:236–244

[7] Voitsekhovskii AV, Nesmelov SN, Dzyadukh SM, Vasil’ev VV, Varavin VS, Dvoretsky SA, Mikhailov NN, Yakushev MV (2015). Admittance of metal-insulator-semiconductor structures based on graded-gap HgCdTe grown by molecular-beam epitaxy on GaAs substrates. Infrared Phys Technol.

[8] Goodwin MW, Kinch MA, Koestner RJ (1988). Metal-insulator-semiconductor properties of HgTe-CdTe superlattices. J Vac Sci Technol A.

[9] Antonov VV, Voitsekhovskii AV (1984) Quantum oscillations of the photo-EMF in an electric field in *p*-Hg_(1-x)_Cd_(x)_Te (*x* of approximately 0.20) with a surface inversion channel. Techn Phys Lett 10:311–313

[10] Yang MJ, Yang CH, Beck JD (1990). Observation of incoherent tunnelling in HgCdTe MIS structure. Semicond Sci Technol.

[11] Bloom I, Nemirovsky Y (1988). Bulk levels and interface calculations for narrow band-gap semiconductors. Sol-St Electron.

